# Variants Fok1 and Bsm1 on VDR are associated with the melanoma risk: evidence from the published epidemiological studies

**DOI:** 10.1186/s12863-015-0163-6

**Published:** 2015-02-11

**Authors:** Wei Hou, Xuefeng Wan, Junwei Fan

**Affiliations:** Department of Dermatology and Venereology, the First Affiliated Hospital of Xinjiang Medical University, Urumqi, 830054 People’s Republic of China

**Keywords:** VDR, Melanoma, Meta-analysis

## Abstract

**Background:**

The vitamin D receptor (VDR) mediates the major cellular activities of vitamin D and regulates various signaling pathways implicated in cancer development and progression. VDR variants have been found associated with the risk of developing melanoma; however, previous epidemiological studies are inconsistent. We have systematically reviewed the published epidemiological literature and conducted a meta-analysis to assess associations between common VDR variants and melanoma risk.

**Results:**

We identified 10 eligible studies that evaluated six VDR variants (Apa1, Bsm1, Cdx2, EcoRV, Fok1, and Taq1) in a total of 4,961 melanoma patients and 4,605 controls. The pooled estimates identified two variants—Fok1 and Bsm1—as significantly associated with melanoma risk, but not for the other four variants Apa1, Cdx2, EcorV and Taq1. For Fok1, the pooled OR was 1.18 (95% CI = 1.06-1.30; I^2^ = 22%) for Ff vs. FF and 1.19 (95% CI = 1.01-1.41; I^2^ = 0%) for ff vs. FF. The dominant genetic model suggested the allele f carriers showed an 18% (pooled OR = 1.18, 95% CI = 1.07-1.29; I^2^ = 0%) increased risk for melanoma compared to homozygote FF. In contrast, the Bsm1 was found to be associated with a decreased risk for melanoma with the pooled OR was 0.85 (95% CI = 0.76-0.95; I^2^ = 0%) for Bb vs. bb and 0.83 (95% CI = 0.68-1.00; I^2^ = 28%) for BB vs. bb. Under the dominant genetic model, a 15% (pooled OR = 0.85, 95% CI = 0.76-0.94; I^2^ = 0%) decrease of melanoma risk was found for those with BB or Bb genotype compared to those of bb genotype.

**Conclusions:**

The VDR variants Fok1 and Bsm1 may influence the susceptibility to developing melanoma, though further studies are needed to verify these conclusions.

## Background

Melanoma is one of the most aggressive skin cancers and is responsible for approximately 75% of skin cancer-related deaths worldwide [1]. An estimated 200,000 new cases and 46,000 deaths occur annually according to a World Health Organization Report. Furthermore, the incidence of melanoma continues to increase in Caucasian populations, with an annual rate of 3–7% [2]. Many risk factors including deranged regulation of susceptibility genes (e.g. CDKN2A, CDK4, MC1R, ATM, and MX2) [3,4], family history of melanoma [5], hair and skin colour [6], socioeconomic status [7] and ultraviolet (UV) light exposure [8] have been identified by previous epidemiological studies. However, the discovery of additional risk factors will aid in providing a more complete understanding of melanoma aetiology.

Vitamin D is a fat-soluble essential vitamin metabolised from 7-dehydrocholesterol in skin cells following UV light exposure or obtained from dietary sources. Vitamin D promotes calcium and phosphate absorption to affect bone metabolism, and plays important roles in the regulation of cellular proliferation, differentiation, apoptosis, migration, and the immune response. Vitamin D deficiency is a worldwide problem, with insufficient vitamin D levels increasing the risk of developing obesity, diabetes, asthma, autoimmune disorders, infectious diseases, and some cancers [9]. Most biological functions of the active form of vitamin D (1,25(OH)_2_D) are mediated by the nuclear vitamin D receptor (VDR), which regulates the transcription of target genes. The VDR gene is located on chromosome 12q12-q14 and comprises 11 exons and 11 introns, with more than 600 single nucleotide polymorphisms having been identified within its coding region [10]. Many epidemiological studies have evaluated associations between VDR variants and various types of cancer including those of the breast, colorectal region, ovary, and prostate [11]. In a mouse model, dysfunctional VDR increased susceptibility to skin cancer following exposure to 7,12-dimethylbenz[a]anthracene (DMBA) [12] or UV light [13]. Given the genetic and environmental interactions between VDR and UV light exposure during skin cancer development, many epidemiological studies have examined associations between VDR variants and melanoma risk. However, the results of these studies have not been consistent. Herein, we aim to clarify associations between VDR variants and melanoma risk via a meta-analysis of relevant published epidemiological studies.

## Methods

The meta-analysis studies were performed following the MOOSE statement.

### Identification of eligible studies

**Figure 1:**
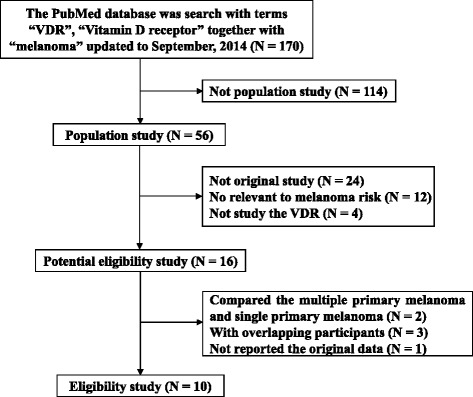
**Working flow chart for the identification of eligible studies.**

Two individuals independently performed a systematic search and comprehensive review of the literature to identify eligible studies. Epidemiological studies deposited in the PubMed database to September, 2014 were searched using the terms “VDR” or “vitamin D receptor” in combination with “melanoma” to identify studies that have reported associations between the VDR variants and melanoma risk. Only the studies that are reported in English were included in the current meta-analysis studies. Following this systematic search, citations within the discovered epidemiological studies and related reviews were checked to identify any missing studies. Figure 1 represents the working flow chart for the identification of eligible studies.

### Inclusion and exclusion criteria

Individual studies that are included in the current meta-analysis studies had to meet the following criteria: 1) determine an association between VDR variant(s) and melanoma risk; 2) be a hospital- or population-based case–control, cross-sectional, or prospective study; 3) provide the genotype distribution within cases and controls (or provide sufficient information to calculate the genotype distribution within participants); 4) the variant was directly genotyped and not imputed; 5) if two or more studies had overlapping participant populations, only the most complete study was included; 6) only those variants with at least three studies examining their association with the melanoma risk were included to reduce potential bias. Studies that did not provide detailed information regarding allele distribution in the cases and controls were excluded. Any study that was reported in language other English was also excluded.

### Data extraction

For each study, two investigators independently extracted the following information: first author’s last name, year of publication, study region, participant ethnicity, origin of controls, number of participants, studied variants, and genotyping methods. For the purposes of meta-analysis, subgroup studies from an individual report were treated as independent studies.

### Statistical analysis

Associations between individual VDR variants and melanoma risk were determined as a pooled odds ratio (OR) together with a 95% confidence interval (CI). Additive and dominant genetic models were applied for each variant and the DerSimonian–Laird method [14] used to calculate pooled estimates under the assumption of a random-effects model that considers heterogeneity both within and between studies. To identify potential bias, the Chi-squared test was used to determine the Hardy–Weinberg equilibrium (HWE) for the genotype distribution in the controls and sensitivity studies were conducted to identify any individual studies that may significantly affect the pooled estimates. Heterogeneity between the studies was measured using the Cochran’s Q-test in combination with the I^2^ statistic, which indicates the percentage of variability across the studies that is caused by heterogeneity rather than by chance alone. A *p* value < 0.05 for the Q-test and/or I^2^ > 25% was recognised as significant and heterogeneity between the studies was noted. Publication bias was assessed graphically through funnel plots and further examined with Egger’s linear regression. Where a significant publication bias was noticed, the trim and fill method was applied. All statistical analyses were performed with STATA 11.0 and Review Manager 5.2. A two-sided *p* value < .05 was considered statistically significant.

## Results

### Characteristics of eligible studies

We identified 10 suitable studies with a total of 4,961 melanoma patients and 4,605 controls which have described associations between common VDR variants and melanoma risk (Table 1) [15-24]. The variants Apa1, Bsm1, Cdx2, EcoRV, Fok1, and Taq1 were each evaluated in at least three studies and were included in the current meta-analysis. Baseline characteristics of the eligible studies are presented in Table 1. Of them, three were performed in the USA, three in UK, one in Spain, one in Italy, one in Poland, and one in Serbia. None of the included studies was derived from the HWE for genotype distribution in the controls, except for one study that was performed by the Zeljic et al. [24], in which a significant difference in the frequency of Apa1 in control subjects was identified (*p* = .001). The study performed by Randerson-Moor et al. [22]. included two independent subgroup studies and these were recognised as individual reports in the current meta-analysis.

**Table 1:** **Characteristics of studies that have determined associations between common VDR variants and melanoma risk**

**Study (Ref)**	**Region**	**Study type**	**Participants**	**SNP site**	**Genotype in cases**	**Genotype in controls**	**Genotyping method**	**HWE-test**
Hutchinson, 2000 [15]	UK	HCC	316 cases, 108 controls	Fok1	105 (FF), 142 (Ff), 45 (ff)	52 (FF), 44(Ff), 12 (ff)	RFLP	0.563
				Taq1	94 (TT), 127 (Tt), 40 (tt)	39 (TT), 41 (Tt), 13 (tt)	RFLP	0.675
Han 2007 [16]	USA	NCC	219 cases, 873 controls	Fok1	77 (FF), 101 (Ff), 37 (ff)	325 (FF), 418 (Ff), 111 (ff)	Taqman	0.193
				Bsm1	85 (bb), 94 (Bb), 29 (BB)	312 (bb), 398 (Bb), 130 (BB)	Taqman	0.869
				Cdx2	132 (GG), 68 (GA), 5 (AA)	548 (GG), 269 (GA), 36 (AA)	Taqman	0.681
Santonocito, 2007 [18]	Italy	PCC	112 cases, 101 controls	Fok1	47 (FF), 41 (Ff), 13 (ff)	41 (FF), 46 (Ff), 14 (ff)	RFLP	0.849
				Bsm1	37 (bb), 54 (Bb), 10 (BB)	26 (bb), 51 (Bb), 24 (BB)	RFLP	0.918
				EcoRV	35 (AA), 51 (AG), 15 (GG)	43 (AA), 45 (AG), 13 GG)	RFLP	0.819
Povey 2007 [17]	UK	PCC	596 cases, 441 controls	EcoRV	196 (AA), 297 (AG), 103 (GG)	130 (AA), 195 (AG), 86 (GG)	RFLP	0.416
Li 2008 [19]	USA	HCC	805 cases, 841 controls	Taq1	330 (TT), 355 (Tt), 120 (tt)	269 (TT), 422 (Tt), 150 (tt)	RFLP	0.485
				Bsm1	305 (bb), 366 (Bb), 134 (BB)	265 (bb), 427 (Bb), 149 (BB)	RFLP	0.308
				Fok1	287 (FF), 427 (Ff), 91 (ff)	344 (FF), 396 (Ff), 101 (ff)	RFLP	0.425
Randerson-Moor 2009 (a, [22] )	UK	PCC	1028 cases, 402 controls	Cdx2	648 (GG), 324 (GA), 56 (AA)	250 (GG), 134 (GA), 18 (AA)	ASPCR	0.993
				EcoRV	337 (AA), 509 (AG), 182 (GG)	137 (AA), 188 (AG), 77 (GG)	ASPCR	0.385
				Fok1	381 (FF), 489 (Ff), 158 (ff)	161 (FF), 176 (Ff), 65 (ff)	ASPCR	0.152
				Bsm1	356 (bb), 497 (Bb), 175 (BB)	134 (bb), 202 (Bb), 66 (BB)	RFLP	0.488
				ApaI	283 (AA), 524 (Aa), 221 (aa)	120 (AA), 190 (Aa), 92 (aa)	ASPCR	0.315
				TaqI	369 (TT), 484 (Tt), 175 (tt)	144 (TT), 194 (Tt), 64 (tt)	RFLP	0.921
Randerson-Moor 2009 (b, [22])	UK	PCC	299 cases, 560 controls	Cdx2	193 (GG), 89 (GA), 17 (AA)	350 (GG), 179 (GA), 31 (AA)	ASPCR	0.204
				EcoRV	87 (AA), 151 (AG), 61 (GG)	198 (AA), 261 (AG), 101 (GG)	ASPCR	0.356
				Fok1	96 (FF), 139 (Ff), 64 (ff)	225 (FF), 255 (Ff), 80 (ff)	ASPCR	0.573
				Bsm1	110 (bb), 145 (Bb), 44 (BB)	134 (bb), 202 (Bb), 66 (BB)	RFLP	0.488
				ApaI	80 (AA), 151 (Aa), 68 (aa)	175 (AA), 283 (Aa), 102 (aa)	ASPCR	0.505
				TaqI	107 (TT), 150 (Tt), 42 (tt)	187 (TT), 273 (Tt), 100 (tt)	RFLP	0.983
Gapska 2009 [20]	Poland	PCC	763 cases, 763 controls	Taq1	315 (TT), 351 (Tt), 94 (tt)	324 (TT), 350 (Tt), 88 (tt)	Taqman	0.657
				Bsm1	327 (bb), 340 (Bb), 96 (BB)	308 (bb), 352 (Bb), 98 (bb)	Taqman	0.869
				Fok1	240 (FF), 377 (Ff), 144 (ff)	252 (FF), 357 (Ff), 143 (ff)	Taqman	0.409
				EcoRV	237 (AA),370 (AG), 154 (GG)	216 (AA), 392 (AG), 147 (GG)	Taqman	0.195
Halsall 2009 [21]	USA	HCC	176 cases, 80 controls	EcoRV	50 (AA),88 (GA), 38 (GG)	34 (AA), 46 (GA), 10 (GG)	RFLP	0.341
Pena-Chilet 2013 [23]	Spain	HCC	530 cases, 314 controls	Taq1	186 (TT), 248 (Tt), 64 (tt)	109 (TT), 141 (Tt), 44 (tt)	Kaspar	0.884
				Fok1	217 (FF), 225 (Ff), 58 (ff)	140 (FF) , 130 (Ff) , 39 (ff)	Kaspar	0.309
				EcoRV	183 (AA), 228 (GA), 94 (GG)	106 (AA), 149 (GA), 45 (GG)	Kaspar	0.531
Zeljic 2014 [24]	Serbia	PCC	117 cases, 122 controls	EcoRV	24 (AA), 66 (GA), 27 (GG)	37 (AA), 51 (GA), 34 (GG)	Taqman	0.071
				Fok1	40 (FF), 60 (Ff), 17 (ff)	46 (FF), 62 (Ff), 14 (ff)	Taqman	0.312
				Taq1	33 (TT), 62 (Tt), 22 (tt)	59 (TT), 48 (Tt), 15 (tt)	Taqman	0.192
				ApaI	55 (AA), 41 (Aa), 21 (aa)	52 (AA), 41 (Aa), 29 (aa)	Taqman	0.001

**Abbreviations:** *ASPCR* allele-specific polymerase chain reaction, *HCC* hospital-based case–control study, *HWE* Hardy–Weinberg equilibrium, *NCC* nested case–control study, *PCC* population-based case–control study, *RFLP* restriction fragment length polymorphism, *SNP* single nucleotide polymorphism.

### Bsm1 and melanoma risk

**Figure 2:**
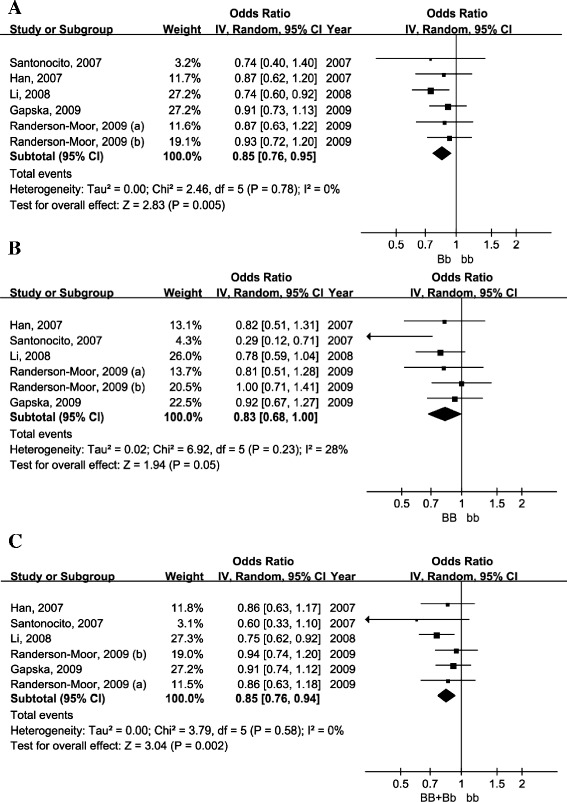
**Forest plot of the association between Bsm1 and melanoma risk for Bb vs. bb (A), BB vs. bb (B) and BB + Bb vs. bb (C).**

Five eligible studies with six subgroups encompassing 3,226 cases and 3,540 controls have previously examined the association between the Bsm1 VDR variant and melanoma risk [16,18-20,22] (Table 1). Calculation of pooled ORs under the random-effects model suggested that Bb carriers had a 15% (pooled OR = 0.85, 95% CI = 0.76–0.95; Figure 2A) decrease in melanoma risk, and BB carriers had a 17% (pooled OR = 0.83, 95% CI = 0.68–1.00; Figure 2B) decrease in melanoma risk when compared with bb genotype individuals. The dominant genetic model also suggested that B allele carriers had a 15% (pooled OR = 0.85, 95% CI = 0.76–0.94; see Figure 2C) decreased risk of melanoma compared with homozygote bb individuals. The heterogeneity test and sensitivity studies suggested the pooled estimates were stable and consistent between the studies (Table 2). No significant publication bias was found for the included studies according to the funnel plot and Egger’s test (Table 3).

**Table 2:** **Summary of results for analysis of associations between VDR variants and melanoma risk (total and stratified analysis)**

**Variant**	**Genetic model**	**Total**				**PCC**				**HCC**			
		**OR (95% CI)**	**Q/df**	**P** ^**a**^	**I** ^**2**^	**OR (95% CI)**	**Q/df**	**P** ^**a**^	**I** ^**2**^	**OR (95% CI)**	**Q/df**	**P** ^**a**^	**I** ^**2**^
Bsm1	Bb vs. bb	0.85 (0.76-0.95)	2.46/5	0.782	0%	0.90 (0.78-1.04)	0.44/3	0.933	0%	0.75 (0.60-0.92)	NA	NA	NA
	BB vs. bb	0.83 (0.68-1.00)	6.92/5	0.227	28%	0.81 (0.59-1.13)	6.55/3	0.088	54%	0.78 (0.59-1.04)	NA	NA	NA
	BB + Bb vs. bb	0.85 (0.76-0.94)	3.79/5	0.580	0%	0.89 (0.78-1.02)	1.98/3	0.577	0%	0.75 (0.62-0.92)	NA	NA	NA
Cdx2	GA vs. GG	0.95 (0.81-1.13)	0.49/2	0.784	0%	0.92 (0.76-1.12)	0.03/1	0.867	0%	NA	NA	NA	NA
	AA vs.GG	1.00 (0.68-1.46)	1.71/2	0.426	0%	1.10 (0.73-1.67)	0.20/1	0.656	0%	NA	NA	NA	NA
	AA + GA vs. GG	0.96 (0.82-1.12)	0.15/2	0.929	0%	0.96 (0.79-1.14)	0.07/1	0.786	0%	NA	NA	NA	NA
EcoRV	GA vs. AA	1.09 (0.93-1.27)	11. 4/7	0.122	39%	1.12 (0.93-1.36)	9.66/5	0.085	48%	1.00 (0.70-1.41)	1.36/1	0.244	26%
	GG vs. AA	1.09 (0.90-1.33)	10.32/7	0.182	32%	1.00 (0.85-1.18)	4.99/5	0.417	0%	1.63 (0.79-3.36)	2.58/1	0.108	61%
	GA + GG vs. AA	1.09 (0.94-1.26)	10.91/7	0.143	36%	1.09 (0.92-1.29)	8.70/5	0.122	43%	1.15 (0.74-1.78)	2.19/1	0.139	54%
Fok1	Ff vs. FF	1.18 (1.06-1.30)	5.65/8	0.686	0%	1.14 (0.99-1.31)	2.21/4	0.697	0%	1.27 (1.08-1.49)	1.62/2	0.444	0%
	ff vs. FF	1.19 (1.01-1.41)	10.25/8	0.248	22%	1.20 (0.92-1.58)	7.05/4	0.133	43%	1.12 (0.85-1.49)	2.39/2	0.303	16%
	ff + Ff vs. FF	1.18 (1.07-1.29)	7.21/8	0.515	0%	1.15 (1.01-1.31)	3.98/4	0.409	0%	1.24 (1.04-1.49)	2.5/2	0.287	20%
Taq1	Tt vs. TT	1.03 (0.83-1.27)	20.47/6	0.002	71%	1.11 (0.86-1.42)	8.06/3	0.045	63%	0.92 (0.64-1.34)	7.66/2	0.022	74%
	tt vs. TT	0.97 (0.75-1.27)	16.07/6	0.013	63%	1.11 (0.78-1.57)	7.98/3	0.046	62%	0.79 (0.57-1.11)	3.26/2	0.196	39%
	tt + Tt vs. TT	1.03 (0.82-1.28)	24.76/6	< 0.001	76%	1.12 (0.86-1.46)	10.20/3	0.017	71%	0.91 (0.63-1.31)	8.23/2	0.016	76%
Apa1	Aa vs. AA	1.14 (0.94-1.39)	0.46/2	0.795	0%	1.14 (0.94-1.39)	0.46/2	0.795	0%	NA	NA	NA	NA
	aa vs. AA	1.07 (0.75-1.54)	3.96/2	0.138	50%	1.07 (0.75-1.54)	3.96/2	0.138	50%	NA	NA	NA	NA
	aa + Aa vs. AA	1.12 (0.93-1.34)	1.68/2	0.431	0%	1.12 (0.93-1.34)	1.68/2	0.431	0%	NA	NA	NA	NA

^a^P for heterogeneity test.

Abbreviations: *OR* odds ratio; *95% CI* 95% confidence interval, *PCC* population-based case–control, *HCC* hospital-based case–control, *NA* not applicable.

**Table 3:** **Egger’s test for publication bias in meta-analysis of the association of six VDR polymorphisms with melanoma risk**

**Variant**	**Egger's test**	**Heterozygote**	**Homozygote**	**Dominant**
Bsm1	t (df)	−0.13 (5)	−2.16 (5)	−0.86 (5)
	P	0.905	0.097	0.439
Cdx2	t (df)	0.56 (1)	−7.66 (1)	0.05 (1)
	P	0.674	0.083	0.97
EcroV	t (df)	2.75 (6)	2.38 (6)	3.38 (6)
	P	0.033	0.054	0.015
Fok1	t (df)	−0.718 (7)	0.72 (7)	−0.15 (7)
	P	0.496	0.493	0.884
Taq1	t (df)	2.57 (5)	1.90 (5)	2.30 (5)
	P	0.05	0.116	0.070
Apa1	t (df)	−3.44 (1)	−0.52 (1)	−1.11 (1)
	P	0.18	0.694	0.467

### Cdx2 and melanoma risk

Two studies from Han et al. [16] and Randerson-Moor et al. [22] examined 1,444 melanoma patients and 1,084 controls to determine the association between the Cdx2 and melanoma risk (Table 1). The pooled estimates under either the additive model or the dominant genetic model suggested no significant association between Cdx2 and melanoma risk (pooled OR = 0.95, 95% CI = 0.81–1.13 for GA vs. GG; pooled OR = 1.00, 95% CI = 0.68–1.45 for AA vs. GG; pooled OR = 0.96, 95% CI = 0.82–1.12 for GA + AA vs. GG; Table 2). Both the Q-test and the I^2^ statistic suggested no significant heterogeneity between the studies, and the Egger’s test indicated no publication bias was present (Tables 2 and 3). No individual study significantly affected the pooled estimates.

### EcoRV and melanoma risk

Seven studies examined a total of 3,621 cases and 2,783 controls to determine the association between the EcoRV VDR variant and melanoma risk [17,18,20-24] (Table 1). No significant associations between the variant and melanoma risk were identified by the meta-analysis, with pooled ORs of 1.09 (95% CI = 0.93–1.27; Q = 11.40, df = 7, *p* = .122; I^2^ = 39%) for the GA vs. AA genotype and 1.09 (95% CI = 0.90–1.33; Q = 10.32, df = 7, *p* = .182; I^2^ = 32%) for the GG vs. AA genotype. Moderate heterogeneity between the studies was detected. No significant change in risk between GA or GG carriers and AA carriers was identified (pooled OR = 1.09, 95% CI = 0.94–1.26; Q = 10.91, df = 7, *p* = .143; I^2^ = 36%) by the dominant genetic model. A forest plot and an Egger’s linear regression test suggested that significant publication bias existed (*p* < .05, Table 3). The trim and fill method was applied to adjust for this, and no significant association between the EcoRV variant and melanoma risk emerged (data not shown).

### Fok1 and melanoma risk

**Figure 3:**
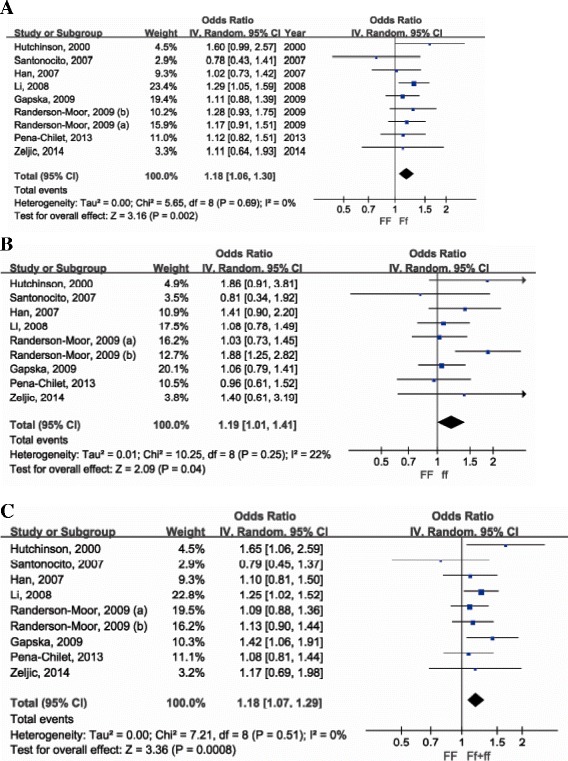
**Forest plot of the association between Fok1 and melanoma risk for Ff vs. FF (A), ff vs. FF (B) and Ff + ff vs. FF (C).**

We identified eight eligible studies with a total of 4,189 cases and 4,084 controls that evaluated the association between the Fok1 VDR variant and melanoma risk [15,16,18-20,22-24] (Table 1). The pooled estimates indicated that an Ff genotype conferred an 18% increase in melanoma risk (pooled OR = 1.18, 95% CI = 1.06–1.30; Figure 3A), whereas ff lead to a 19% increased risk of melanoma (pooled OR = 1.19, 95% CI = 1.01–1.41; Figure 3B) when compared with those of the FF genotype. Carriers of the f allele had an 18% (pooled OR = 1.18, 95% CI = 1.07–1.29; Figure 3C) increased risk of melanoma compared with homozygote FF individuals. There was no significant heterogeneity between the studies according to the Q-test and the I^2^ value; whereas sensitivity studies indicated that the pooled estimates were stable and no individual study significantly affected the pooled estimates (Tables 2 and 3).

### Taq1 and melanoma risk

Six studies incorporating 3,858 cases and 3,110 controls investigated the association between the Taq1 VDR variant and melanoma risk [15,19,20,22-24] (Table 1). The pooled OR under the assumption of random-effects model were 1.03 (95% CI = 0.83–1.27) for Tt vs. TT and 0.97 (95% CI = 0.75–1.27) for tt vs. TT genotype, suggested that the variant may not be the susceptibility factor for melanoma (Table 2). A null association for Taq1 and melanoma risk (pooled OR = 1.03, 95% CI = 0.82–1.28; Table 2) was observed under the dominant genetic model. The Q-test and I^2^ statistic suggested that significant heterogeneity was present between the included studies. No individual study significantly affected the overall estimates, and no significant publication bias was detected as suggested by the Egger’s test (*p* > .05, Table 3).

### Apa1 and melanoma risk

Two studies that performed by the Randerson-Moor et al. [22] and the Zeljic et al.[24] have examined the association between the Apa1 variant and melanoma risk in a total of 1,444 cases and 1,084 controls. The pooled ORs under the random-effects model were 1.04 (95% CI = 0.94–1.39) for Aa vs. AA and 1.07 (95% CI = 0.75–1.54) for aa vs. AA (Table 2). No significant heterogeneity between the studies was identified (Table 2). Under the assumptions of the dominant model, Aa and aa carriers also showed no significant increased risk for melanoma compared with AA carriers (pooled OR = 1.12, 95% CI = 0.93–1.34; *p* for Q-test = .431, I^2^ = 0%; Table 2). The sensitivity studies suggested that the overall estimate was not significantly affected by any individual study. No significant publication bias was found for the studies according to the Egger’s test (Table 3).

## Discussion

Following a systematic review and meta-analysis of relevant published epidemiological studies to date, we have identified that the VDR variants Bsm1 and Fok1 are associated with the risk of developing melanoma, while four other variants (Apa1, Cdx2, EcoRV, and Taq1) are not. As a nuclear receptor phosphoprotein, VDR binds to its ligand 1,25(OH)_2_D with high affinity and regulates the expression of target genes through zinc finger-mediated DNA binding and protein–protein interactions [25]. Signalling pathways downstream of VDR are involved with the regulation of cellular proliferation, differentiation, and apoptosis. VDR is present in normal skin keratinocytes and skin cancer cells derived from malignant melanomas and squamous cell carcinomas. In a mouse model, topical application of 1,25(OH)_2_D could inhibit DMBA-induced skin carcinogenesis [26], and VDR knockout mice were more susceptible to DMBA-induced skin tumourigenesis [12]. Moreover, exposure to UV light, particularly UVB, is a major environmental risk factor for melanoma, and VDR knockout mice developed melanoma more rapidly and with greater penetrance than did wild-type mice following UV exposure [13]. Taken together, these results indicate that dysfunctional VDR signalling pathways are implicated in skin cancer development and progression.

Fok1 is located in exon 2 of the VDR coding region. The F to f transition alters the translation start site for the VDR protein, making the f allele three amino acids longer. This larger VDR molecule is less active than the regular-sized receptor following stimulation with 1,25(OH)_2_D [27]. Moreover, VDR protein encoded by the F allele is more stable than the f isoform and is more effective in suppressing the oestrogen receptor signalling pathway and other pro-inflammatory pathways in breast cancer cells [28]. Etten et al. reported that the Fok1 variant affects immune system regulation, with the shorter VDR molecule enhancing NF-κB and NFAT-driven transcription and stimulating higher IL-12p40 promoter-driven transcription activity in the absence of 1,25(OH)_2_D compared with the longer VDR isoform [29]. Additionally, human monocytes and dendritic cells with an FF genotype express higher level of IL-12 compared with those of an ff genotype, and lymphocytes with an FF genotype proliferate more strongly following phytohemagglutinin stimulation [29]. Whereas some uncertainty remains, several epidemiological studies have suggested that the Fok1 variant is a susceptibility factor for both breast [30] and ovarian cancers [31]. Although no significant association between Fok1 and the risk of melanoma has been identified by the majority of relevant published studies, this may result from low statistical power. The current meta-analysis has identified that the f allele may be associated with increased risk of melanoma, with this association being observed in both population-based and hospital-based case–control studies. Together, these data suggest that the Fok1 variant may be a susceptibility factor for melanoma risk.

Bsm1 is located within the 3′-UTR region of VDR and is in high linkage disequilibrium with Apa1 and Taq1. Previous studies identified the baT and Bat haplotypes as the most frequent in the population, with baT mRNA having lower stability than Bat transcripts [32]. Chudek et al. reported that individuals with a BB genotype had lower plasma 1,25(OH)_2_D levels compared with bb individuals [33]. We report here that the Bsm1 variant is associated with decreased risk of melanoma, whereas two other variants—Apa1 and Taq1—are not associated with melanoma risk. Liu et al. recently reported no significant association between Bsm1, Apa1, and Taq1 variants and ovarian cancer through meta-analysis of up-to-date epidemiological studies [31], while Xu et al. identified that the VDRvariants is associated with colorectal cancer [11]. Xu et al. also found that the Taq1 variant was associated with decreased prostate (but not colorectal, breast, or ovarian) cancer risk [11]. These divergent associations between variants and risk of different cancers may be owing to cancer-specific genetic backgrounds, which may alter the actions of vitamin D in different tissues. These variants may also be in high linkage disequilibrium with other functional variants that may confer associations between the variants and risk of different types of cancer. We identified a protective effect of the Bsm1 variant for melanoma in a hospital-based case–control study, but not in a population-based case–control study. Therefore, larger studies are warranted to elucidate the relationship between these variants and melanoma risk.

We detected a high linkage disequilibrium between the Cdx2 and EcoRV variants, both of which are located in the *VDR* promoter region where they may influence gene transcription [17]. The G to A transition of the Cdx2 variant has been found to enhance the binding capacity of the intestinal-specific transcription factor CDX2 [34]. Our current meta-analysis identifies no significant association between the Cdx2 variant and melanoma risk. This may result from a lack of CDX2 expression by skin cells, or simply be the result of a small sample size with low statistical power. EcoRV is located within the core sequence of a putative glutamyl-transfer RNA amidotransferase subunit A 3 (GATA-3) binding site, and the A allele exhibits decreased GATA-3 binding capacity compared with the G allele [35]. GATA-3 is reportedly involved in the differentiation of T-helper 2 lymphocytes, which play important roles in immune system responses [36]. Our current meta-analysis identifies no significant association between the EcoRV variant and melanoma risk. These data indicate that the Cdx2 and EcoRV variants are not susceptibility factors for melanoma.

There are several limitations to our current meta-analysis. First, the sample size used to determine associations between individual variants and melanoma risk was relatively small. Therefore, the lack of an association between some variants and melanoma risk may result from low statistical power. Second, nearly all of the eligible studies were performed in Western countries, and whether the associations identified are present in other populations remains unknown. Third, the method we employed did not take into account other co-factors such as age, sex, smoking, vitamin D level, or the latitude at which participants reside, all of which may modify the associations between specific VDR variants and melanoma risk.

## Conclusions

This meta-analysis identified that the VDR variants Bsm1 and Fok1 are significantly associated with melanoma risk, whereas four other variants—Apa1, Cdx2, EcoRV, and Taq1—are not. Nevertheless, more studies are warranted to validate these results and further understand the mechanisms underlying these associations.

## Abbreviations

VDR: Vitamin D receptor
OR: Odds ratio
95% CI: 95% confidence interval
HWE: Hardy–Weinberg equilibrium
UV: Ultraviolet
